# Astaxanthin Prevented Acute Alcoholic Cardiomyopathy by Maintenance of Mitophagy‐Mediated Mitochondrial Homeostasis

**DOI:** 10.1111/jcmm.70714

**Published:** 2025-07-13

**Authors:** Guoyong Fan, Tinghao Liu, Xuemei Chen, Yan Guo, Xuan Li, Yue He, Peng Hua, Xiufei Lin, Dini Lin, Xiaoqing Yan, Shicui Jiang, Chi Zhang

**Affiliations:** ^1^ Wenzhou Key Laboratory for the Diagnosis and Prevention of Diabetic Complications, the Third Affiliated Hospital of Wenzhou Medical University (Ruian People's Hospital) Ruian China; ^2^ Liji Medical Research Institute (Life and Health Research Institute, the Third Affiliated Hospital of Wenzhou Medical University) Wenzhou China; ^3^ Ruian Center of Chinese‐American Research Institute for Diabetic Complications, The Third Affiliated Hospital of Wenzhou Medical University (Ruian People's Hospital) Wenzhou China; ^4^ School of Pharmaceutical Sciences, Wenzhou Medical University Wenzhou China; ^5^ Department of Endocrinology The Third Affiliated Hospital of Wenzhou Medical University (Ruian People's Hospital) Wenzhou China

**Keywords:** acute alcoholic cardiomyopathy, astaxanthin, mitochondrial homeostasis, mitophagy, oxidative stress

## Abstract

Acute alcoholism commonly targets the myocardium, triggering acute alcoholic cardiomyopathy (ACM). Strong evidence suggested that mitochondrial dysfunction‐induced myocardial oxidative stress is involved in the subcellular pathogenesis of acute ACM. We investigated whether astaxanthin (AST), an antioxidant lutein carotenoid, prevents acute ACM and explored the underlying mechanisms. C57BL/6J mice were used to model ethanol‐induced ACM and were treated with AST (100 mg/kg/day) alongside the autophagy inhibitor, 3‐methyladenine (10 mg/kg/day). Cardiac function, heart pathology, cardiac hypertrophy, cardiomyocyte apoptosis, oxidative stress and mitochondrial function were evaluated, respectively in response to ethanol and/or AST. The in vivo study showed that ethanol‐induced cardiac dysfunction, morphological injury, cardiomyocyte apoptosis and oxidative stress were mitigated by AST. AST's anti‐apoptotic effects against ethanol were confirmed in vitro. Ethanol‐induced cardiac apoptosis is closely associated with mitochondrial dysfunction which was attenuated by AST characterised by inhibiting fission and promoting fusion, as well as maintaining stable mitochondrial membrane potential, increased ATP production, enhanced biogenesis and restored mitophagy. Autophagy inhibition suppressed AST‐induced myocardial protection, indicating that myocardial mitophagy mediates AST effects. The present study demonstrates that AST induces cardiac protection against acute ACM by improving cardiac function, reducing pathological changes, and inhibiting oxidative stress, inflammation and apoptosis through preserved myocardial mitophagy‐mediated mitochondrial homeostasis.

## 1 Introduction

The consumption of alcoholic beverages, integral to social interactions, has rapidly increased [1, 2]. However, excessive intake can lead to severe myocardial damage, known as alcoholic cardiomyopathy (ACM) [1, 2]. ACM can be categorised into chronic and acute forms according to the onset time and the pathological characteristics. The acute form is marked by diminished cardiac cell contraction, left ventricular dilation, heart failure, arrhythmias and myocardial inflammation [3]. Strong evidence shows that oxidative stress plays a significant role in the onset and progression of acute ACM [4, 5, 6]. Moreover, studies have further demonstrated that alcohol‐induced mitochondrial injury is a key contributor to myocardial oxidative stress and the subsequent acute ACM development. Autopsy findings in patients with ACM have highlighted prominent mitochondrial disorders, increased lysosome numbers and mitochondrial alterations [7]. In vivo studies further support this, demonstrating that excessive alcohol intake leads to severe cardiomyocyte mitochondrial dysfunction, characterised by pore opening in the mitochondrial membrane, reduced membrane potential and disrupted ATP production [8, 9, 10, 11]. These mitochondrial abnormalities contribute to myocardial oxidative stress and subsequent ACM development. Efficient and safe inhibition of myocardial mitochondrial dysfunction and oxidative stress is a promising strategy for preventing acute ACM.

Astaxanthin (AST, 3,3′‐dihydroxy‐β, β‐carotene‐4,4′‐dione), a compound belonging to the xanthophyll carotenoids family, is known for its potent antioxidant properties [12, 13]. Several studies have highlighted its ability to prevent various health issues, including tumour development, nerve injury, hypertension and diabetic complications, by combating oxidative stress [14, 15, 16, 17, 18]. Its widespread safety profile has led to its extensive use in food additives, aquaculture, cosmetics and health products [19, 20]. Recent research has shed light on AST's benefits to heart health. In vitro studies have demonstrated that AST administration suppressed cardiomyocyte injury induced by oxidants or hypoxia/reoxygenation, implying that AST can directly act on cardiomyocytes to exert its function [21, 22, 23]. Notably, a pilot clinical study demonstrated that a 3‐month AST supplementation improved cardiac function in patients with heart failure and left ventricular systolic dysfunction [24]. Our previous studies have demonstrated AST's efficacy in preventing chronic ACM by suppressing endoplasmic reticulum stress‐mediated cardiomyocyte apoptosis. However, due to different pathogeneses between chronic and acute ACM, the preventive effects of AST on acute ACM remain to be explored [25]. Mounting evidence suggests that the cardioprotective effects of AST are closely linked to its ability to maintain myocardial mitochondrial function [26, 27, 28]. Moreover, studies have demonstrated that AST mitigates cardiotoxicity induced by substances such as homocysteine or isoproterenol by inhibiting mitochondrial dysfunction and oxidative damage [29, 30]. As known in common, autophagy, especially mitophagy, plays an important role in maintaining mitochondrial homeostasis [31, 32, 33]. Furthermore, strong evidence indicated that AST induced beneficial effects on dry eye disease, diabetic kidney injury and renal ischemia–reperfusion damage via preserving the stabilisation of autophagy [34, 35, 36]. Importantly, Cui et al. reported that AST protects vertebral cartilage endplate against oxidative stress and degeneration by promoting mitophagy via activation of the Nrf‐2/HO‐1 pathway [37]. In contrast, Deuster et al. found that AST protects against heat‐induced mitochondrial alterations in the mouse hypothalamus by reducing excessive mitophagy [38]. Thus, the paradoxical findings above imply that the effect of AST on mitophagy is to maintain its stabilisation rather than simply upregulate or downregulate its level.

Therefore, in the present study, we focused on identifying whether AST induces a preventive effect on acute ACM. If so, additionally, we aimed to clarify the contribution of mitophagy‐mediated maintenance of mitochondrial homeostasis to AST‐induced myocardial protection against acute alcohol exposure.

## 2 Results

### 2.1 AST Prevented Acute Alcohol Treatment‐Induced Cardiac Dysfunction and Morphological Alterations in C57BL/6J Mice

**FIGURE 1:**
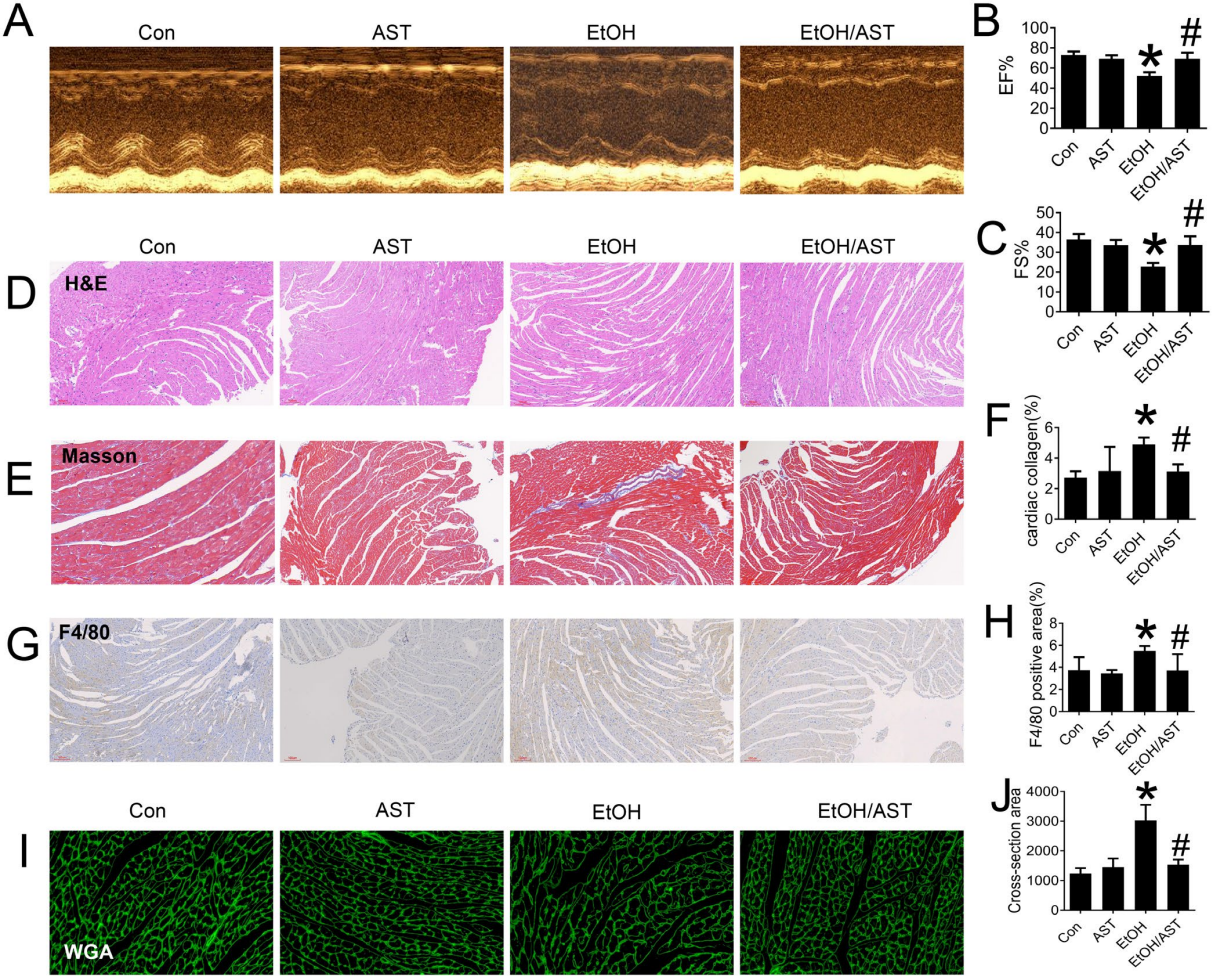
AST prevented acute alcohol treatment‐induced cardiac dysfunction and morphological alterations in C57BL/6J mice. Echocardiography was performed on mice in each group to evaluate the cardiac systolic function (A–C). Cardiac morphological changes were detected by HE staining (D). Cardiac collagen accumulation and fibrosis were evaluated by Masson staining (E, F). Cardiac inflammation was evaluated by F4/80 staining (G, H). Cardiac hypertrophy was evaluated by WGA staining (I, J). Data are presented as the mean ± standard deviation (*n* = 3‐6/group). *p < 0.05 versus Con group; ^#^ *p* < 0.05 versus EtOH group.

Echocardiography results demonstrated that acute alcohol exposure led to diminished ejection fraction percentage (%EF) and fractional shortening (%FS, Figure 1A–C). These findings indicate that acute alcohol exposure induces cardiac dysfunction. However, the reduced %EF and %FS were improved after treatment with AST (Figure 1A–C), suggesting that AST induced a beneficial effect on cardiac systolic function against acute alcohol exposure.

Cardiac dysfunction is closely linked to the changes in cardiac morphology. In the present study, we observed abnormal cardiac structures, including disorganised myocardial fibres, myocardium loss, myofibril breakage and inflammatory cell infiltration (Figure 1D). Masson's trichrome staining showed disorganised collagen fibres, increased blue extracellular matrix components, and significant collagen accumulation with interconnections in a meshwork (Figure 1E,F); F4/80 staining showed excessive mature macrophages accumulated in myocardium tissue (Figure 1G,H), indicating the acute alcohol intake induced cardiac inflammatory effect. Additionally, cardiomyocyte enlargement indicating cardiac hypertrophy was also observed in acute alcohol‐treated mice measured by WGA staining (Figure 1I,J). Conversely, AST treatment prevented these abnormal morphological changes in response to acute alcohol intake (Figure 1D–J), suggesting that AST's protective effect on cardiac function against acute alcohol intake may be due to the preservation of a normal cardiac structure as well as suppression of cardiac fibrosis, inflammation and hypertrophy.

### 2.2 AST Prevented Acute Alcohol Treatment‐Induced Cardiac Apoptosis In Vivo and In Vitro

**FIGURE 2:**
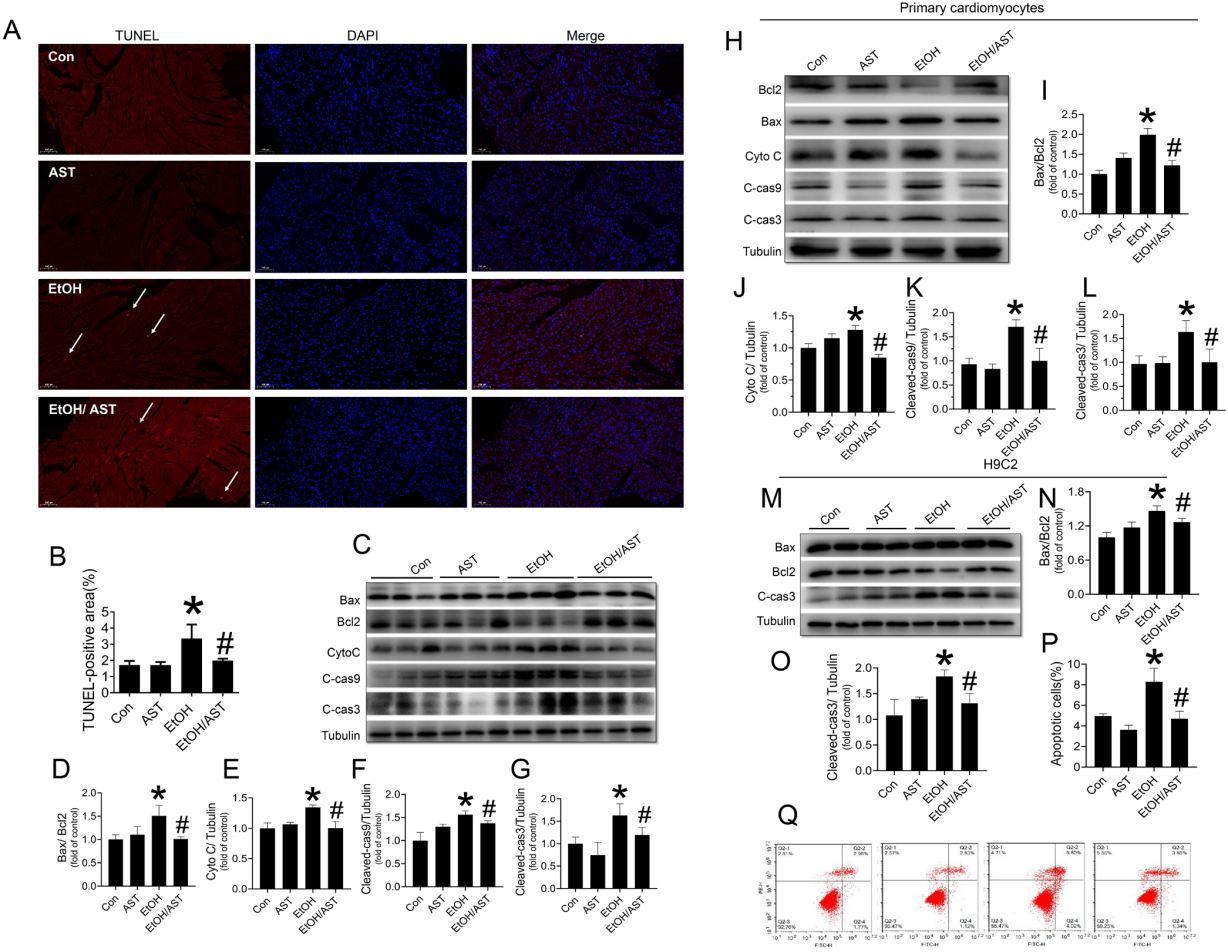
AST prevented acute alcohol treatment‐induced cardiac apoptosis in vivo and in vitro. Results from in vivo studies: Apoptosis in cardiac tissues was observed by TUNEL fluorescence staining (A, B). The apoptotic markers including Bax/Bcl‐2 ratio (C, D), CytoC level (C, E), C‐cas9 level (C, F) and C‐cas3 level (C, G), were measured by Western‐blot assay. Results from in vitro studies: The apoptotic markers including Bax/Bcl‐2 ratio (H, I), CytoC level (H, J), C‐cas9 level (H, K) and C‐cas3 level (H, L) in alcohol and/or AST treated primary cardiomyocytes, were measured by Western‐blot assay. The apoptotic markers including Bax/Bcl‐2 ratio (M, N), and C‐cas3 level (M, O) in alcohol and/or AST treated cardiac H9c2 cells, were also measured by Western‐blot assay. Moreover the apoptosis in H9c2 cells was also measured by flow cytometry (P, Q). Four groups of animal experiments were performed in vivo study. Three independent experiments were performed in vitro study. Data are presented as the mean ± standard deviation (*n* = 3‐6/group). *p < 0.05 versus Con group; ^#^ *p* < 0.05 versus EtOH group.

In the present study, TUNEL staining revealed that apoptotic cells were rarely observed in the cardiac tissues of non‐alcohol‐treated mice. However, acute alcohol treatment significantly induced cardiac cell apoptosis, as indicated by an increase in TUNEL‐positive cells (red). This alcohol‐induced cardiac apoptosis was strongly attenuated by AST treatment (Figure 2A,B). Additionally, western blot analysis showed that the ratio of Bax to Bcl‐2 (Figure 2C,D) and the expression of several apoptotic markers, including CytoC (Figure 2C,E), C‐cas9 (Figure 2C,F) and C‐cas3 (Figure 2C,G), were upregulated in response to acute alcohol treatment. Notably, AST attenuated the protein level of these apoptotic markers, indicating its anti‐apoptotic effect against acute alcohol treatment (Figure 2).

To determine whether the anti‐apoptotic effect of AST in response to acute alcohol treatment was directed towards cardiomyocytes, both the cardiac cell line was utilised in an in vitro study. Alcohol exposure significantly induced apoptosis in primary cardiomyocytes of C57BL/6J mice, shown by the upregulation of multiple apoptotic markers, including the Bax/Bcl‐2 ratio (Figure 2H,I), CytoC (Figure 2H,I), C‐cas9 (Figure 2H,K) and C‐cas3 (Figure 2H,L). Similar changes in the indices above were also observed in alcohol‐treated H9c2 cells (Figure 2M–O). Additionally, flow cytometric analysis further confirmed that alcohol treatment induced apoptosis in H9c2 cells (Figure 2P,Q). However, AST treatment significantly inhibited apoptosis in both primary cardiomyocytes and H9c2 cells (Figure 2), indicating that AST can directly act on cardiomyocytes to prevent alcohol‐induced cardiomyocyte apoptosis.

### 2.3 AST Prevented Acute Alcohol Treatment‐Induced Cardiac Oxidative Stress Both In Vivo and In Vitro

**FIGURE 3:**
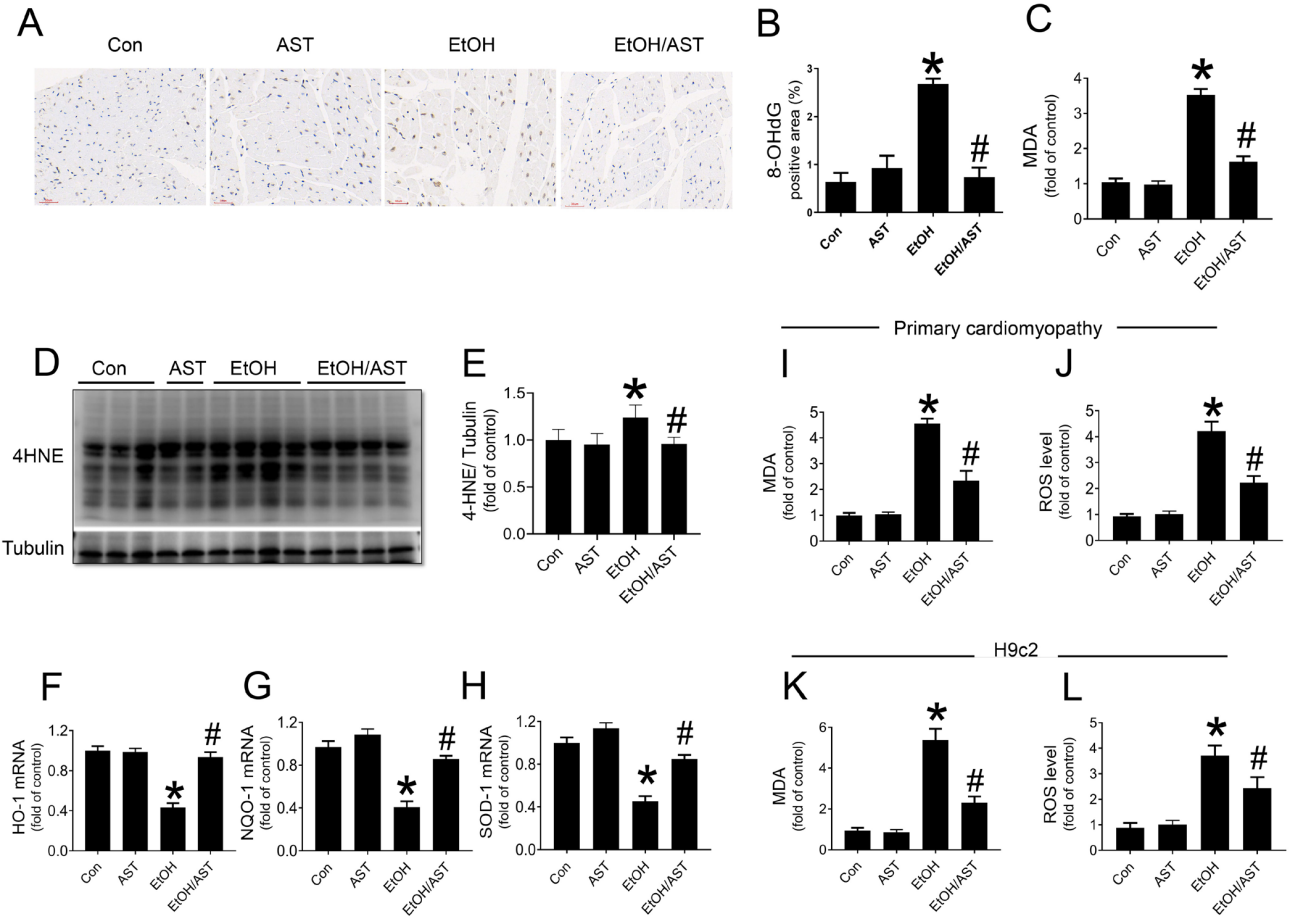
AST prevented acute alcohol treatment‐induced cardiac oxidative stress both in vivo and in vitro. Results from in vivo studies: Oxidative stress in myocardium was measured by 8‐hydroxydeoxyguanosine (8‐OhDG) staining (A, B) and the detection of MDA level (C), which is one of end‐products of lipid peroxidation. Western‐blot analysis was applied to detect the expression level of 4‐HNE, a classic marker for oxidative stress (D, E). The levels of HO‐1, NQO‐1, SOD‐1 in mice were assessed using RT‐qPCR (F, G). Results from in vitro studies: The level of MDA (I, K) and ROS (J, L) in both primary cardiomyocytes and H9c2 cells were detected by ELISA or lucigenin‐enhanced chemiluminescence. Four groups of animal experiments were performed in vivo study. Three independent experiments were performed for the in vitro study. Data are presented as the mean ± standard deviation (*n* = 3‐7/group). *p < 0.05 versus Con group; ^#^ *p* < 0.05 versus EtOH group.

8‐OhDG staining revealed a robust activation of oxidative stress in the myocardium following acute alcohol treatment (Figure 3A,B). Additionally, increased levels of MDA (Figure 3C) and the upregulation of the classic oxidative stress marker, 4‐HNE (Figure 3D,E), further confirmed the induction of cardiac oxidative stress. Moreover, we also observed a reduction in the expression of multiple antioxidants at the mRNA level, including *ho‐1*, *nqo‐1* and *sod‐1* in response to alcohol treatment (Figure 3F–H), suggesting that the induction of cardiac oxidative stress by acute alcohol treatment could be attributed to impaired antioxidative capability. Additionally, in vitro studies further demonstrated that alcohol treatment induced oxidative stress in both primary cardiomyocytes and H9c2 cells, as evidenced by increased MDA and reactive oxygen species (ROS) levels (Figure 3I–L). In contrast, treatment of AST significantly attenuated alcohol‐induced cardiac oxidative stress by rebalancing the redox (Figure 3).

### 2.4 AST Protects Mitochondrial Function in Cardiac Cells Against Alcohol‐Induced Damage In Vitro and In Vivo

**FIGURE 4:**
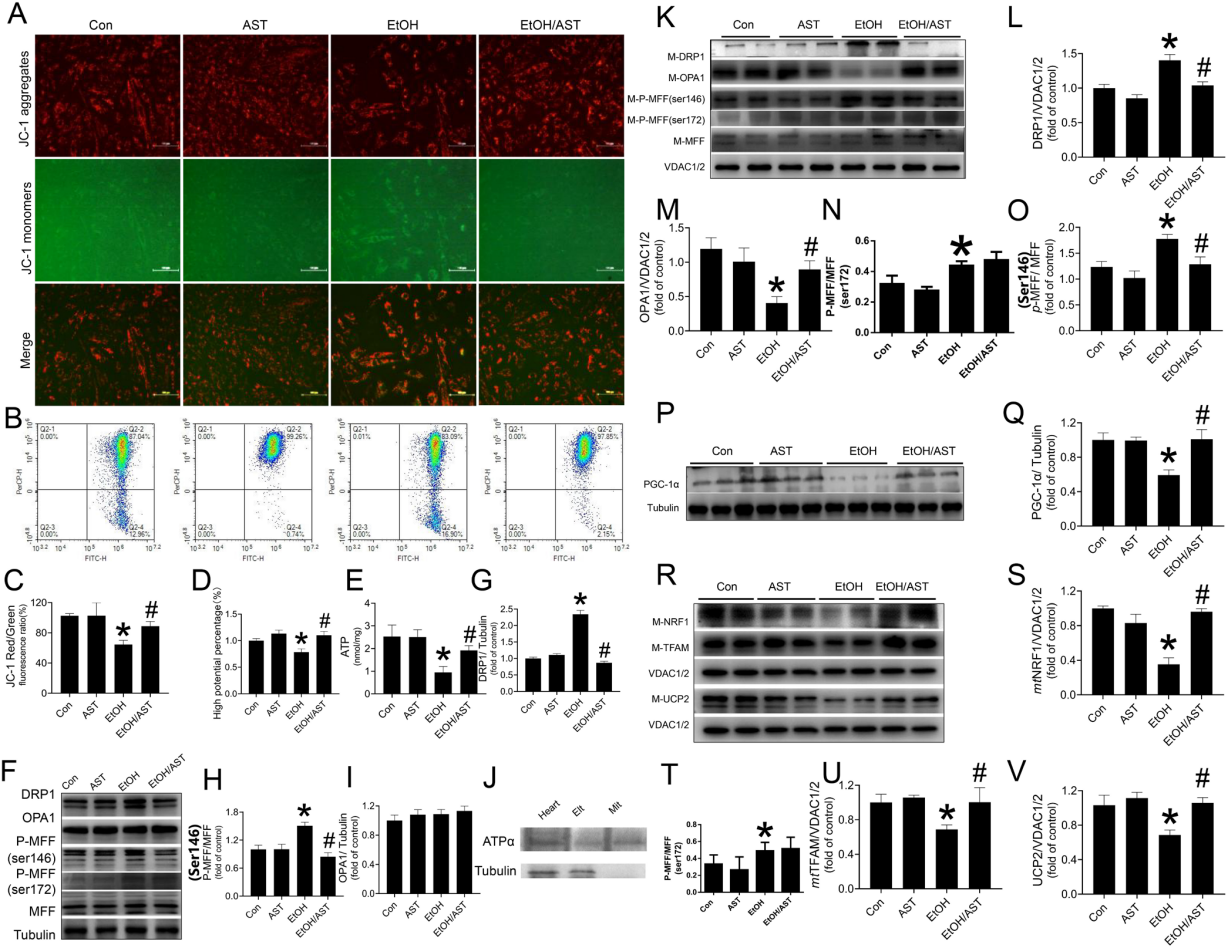
The effect of AST on cardiac mitochondrial function under alcohol exposure was evaluated in vitro and in vivo. Results from in vitro studies: Mitochondrial membrane potential (MMP) as measured by JC‐1 staining (A, C) and flowcytometry (B, D). The change of ATP content in primary cardiomyocytes was measured using a luciferase bioluminescence‐based ATP assay kit (E). Western‐blot analysis was applied to detect the expression level of DRP1 (F, G), OPA1 (F, I) and phosphorylated MFF (Ser146) (F, H), phosphorylated MFF (Ser172) (F, T). Results from in vivo studies. Mitochondrial purification marker protein ATPα, Tublin were measured by Western‐blot assay, Elt, first elution through the magnetic column; Heart, heart homogenate; Mit, isolated mitochondria (J). The levels of mitochondrial fission and fusion markers including mitochondrial DRP1 (*m*‐DRP1; K, L), OPA1 (*m*‐OPA1; K, M), phosphorylated MFF (Ser146) (*m*‐*p*‐MFF; K, O) phosphorylated MFF (Ser172) (*m*‐*p*‐MFF; K, N)were measured by Western‐blot assay. The levels of PGC‐1α (P, Q), mitochondrial NRF1 (*m*‐NRF1; R, S), mitochondrial TFAM (m‐TFAM; R, U) and mitochondrial UCP2 (*m*‐UCP2; R, V), which represent mitochondrial biogenesis were also measured by Western‐blot assay. Three independent experiments were performed in vitro study. Four groups of animal experiments were performed in vivo study. Data are presented as the mean ± standard deviation (*n* = 3‐12/group). *p < 0.05 versus Con group; ^#^ *p* < 0.05 versus EtOH group.

Given the pivotal role of mitochondrial dysfunction in precipitating cardiac oxidative stress and subsequent apoptosis, the effect of AST on cardiac mitochondrial function under alcohol exposure was evaluated in vitro. JC‐1 staining revealed severe mitochondrial membrane depolarisation in primary cardiomyocytes following alcohol treatment characterised by the ratio decrease of JC‐1 aggregates to monomers (Figure 4A,C). This alcohol‐induced decline in mitochondrial membrane potential was further corroborated by flow cytometry analysis (Figure 4B,D). However, the impaired mitochondrial potential induced by alcohol exposure was notably prevented in response to AST (Figure 4A–D). Moreover, AST also preserved the reduction in ATP content in primary cardiomyocytes in response to alcohol treatment (Figure 4E). Maintaining a balance between mitochondrial fusion and fission is crucial for sustaining mitochondrial homeostasis and function. Our findings revealed a significant upregulation of mitochondrial fission in primary cardiomyocytes upon alcohol treatment, characterised by increased expression of DRP1 (Figure 4F,G), phosphorylated and non‐phosphorylated MFF (Figure 4F,H,T)，and it was found that P‐MFF expression was increased in the model group, whereas after the administration of the intervention, astaxanthin was found to exert its ameliorating effect through phosphorylation site 146 (Figure 4F,H,T). However, alcohol treatment did not affect mitochondrial fusion, as evidenced by unchanged OPA1 expression (Figure 4F,I). Analysis of the purity of the mitochondrial fraction by western blot showed that the isolated mitochondria had higher levels of ATP synthase α in comparison with protein fractions obtained from the heart or first centrifugation to separate the supernatant. In contrast, the cytoplasmic (tubulin‐α) components were not detected in the mitochondrial fraction (Figure 4J). In vivo study also confirmed the inhibitory effect of AST on excessive mitochondrial fission in the myocardium induced by acute alcohol exposure (Figure 4K,L,N,O). However, contrary to the findings in the in vitro study, acute alcohol treatment decreased, but AST preserved normal cardiac mitochondrial OPA1 level (Figure 4K,M).

Additionally, we also found that AST protected cardiac PGC‐1α‐mediated mitochondrial biogenesis characterised by reserving the expression of PGC‐1α (Figure 4P,Q), and its downstream targets including NRF1 (Figure 4R,S), TFAM (Figure 4R,U) and UCP2 (Figure 4R,V). The findings above suggested that AST prevented acute alcohol treatment‐induced cardiac mitochondrial injury and might be attributed to its maintenance of mitochondrial homeostasis by improving mitochondrial fusion, fission and biogenesis.

### 2.5 AST Improved Cardiac Mitophagy in Response to Acute Alcohol Treatment

**FIGURE 5:**
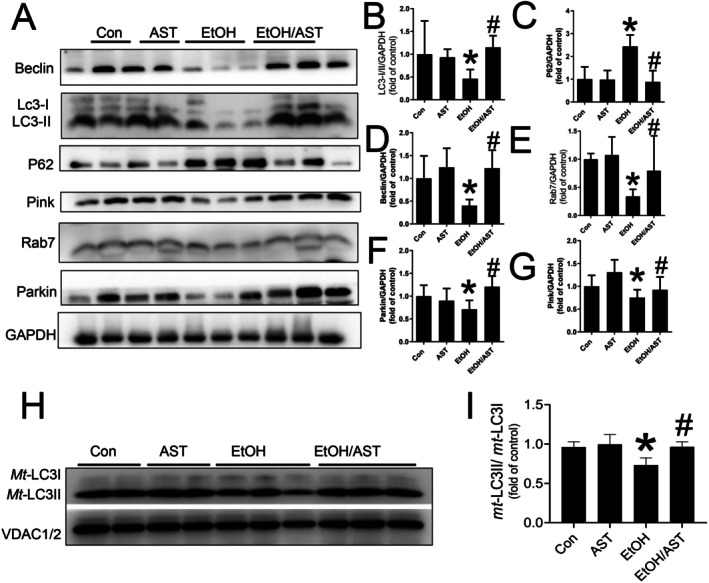
AST improved cardiac mitophagy in response to acute alcohol treatment. LC3II/I ratio (A, B), the expressions of Beclin (A, D), Rab7 (A, E) and P62 (A, C), as well as the markers of mitophagy including Pink (A, F) and Parkin (A, G), were detected by Western‐blot assay. Isolated mitochondrial proteins were used to detect the expression level of mitochondrial ratio of LC3II to LC3I (H, I). Data are presented as the mean ± standard deviation (*n* = 3–6/group). *p < 0.05 versus Con group; ^#^ *p* < 0.05 versus EtOH group.

Strong evidence has demonstrated that mitochondrial homeostasis, including biogenesis, fusion and fission, is regulated by mitophagy. In the present study, we found that acute alcohol treatment significantly impaired myocardial autophagy characterised by a decrease in the LC3II/I ratio (Figure 5A,B), as well as the expression of Beclin1 (Figure 5A,D) and Rab7 (Figure 5A,E), and an increase in P62 expression (Figure 5A,C). However, the autophagy disorder was prevented once co‐treated with AST (Figure 5A–E). Importantly, mitophagy markers, such as Pink and Parkin, were also downregulated (Figure 5A,F,G), indicating that the reduction in myocardial autophagy induced by acute alcohol might be due to impaired cardiac mitophagy. However, the reduced Pink and Parkin levels were significantly prevented by administration of AST (Figure 5A,F,G). Moreover, the beneficial effect of AST on myocardial mitophagy was further confirmed by the increased *mt*‐LC3II/I ratio compared to the acute alcohol treatment (Figure 5H,I). This finding suggests that the protective effect of AST on myocardial mitochondrial homeostasis might be closely related to the maintenance of mitophagy.

### 2.6 AST Prevented Acute ACM by Maintaining Cardiac Mitophagy

**FIGURE 6:**
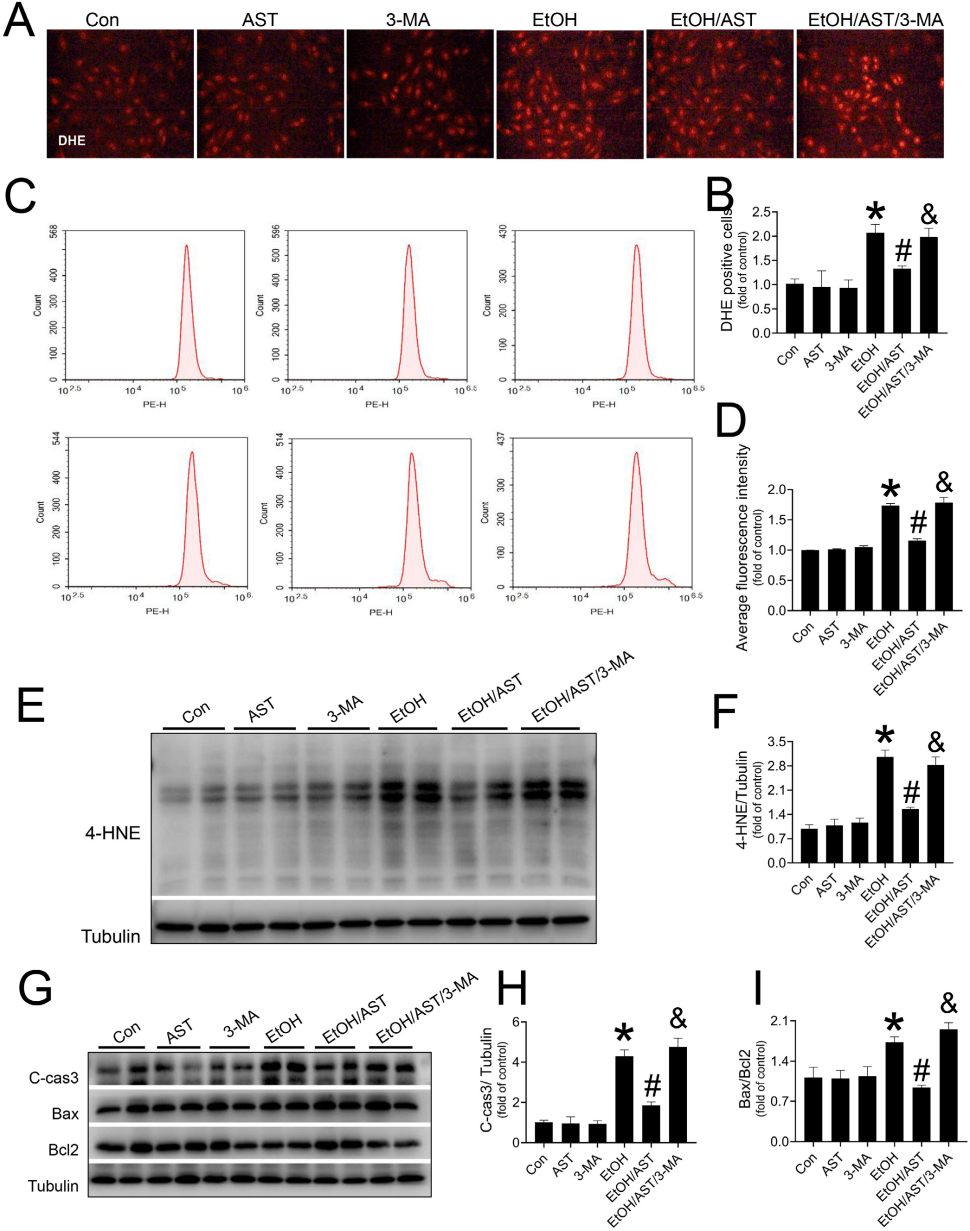
The impact of 3‐MA on AST‐induced anti‐oxidative effect against alcohol treatment. DHE was stained to evaluate the oxidative stress (A, B), which was also confirmed by flow cytometry (C, D). Western‐blot analysis was applied to detected the oxidative marker 4‐HNE (E, F) and the apoptotic makers including C‐cas3 (G, H) and Bax/Bcl‐2 ratio (G, I). Three independent experiments were performed in this study. Data are presented as the mean ± standard deviation (*n* = 3/group). *p < 0.05 versus Con group; ^#^ *p* < 0.05 versus EtOH group; ^&^ *p* < 0.05 versus EtOH/AST group.

To explore the role of mitophagy in AST's preventive effects in mice exposed to acute alcohol treatment, we used the autophagy inhibitor 3‐MA in vitro studies. In vitro studies confirmed that AST administration significantly prevented oxidative stress in alcohol‐treated H9c2 cells by reducing ROS level as detected by DHE staining (Figure 6A,B), flow cytometry (Figure 6C,D) and western blotting for 4‐HNE (Figure 6E,F). Additionally, alcohol‐induced C‐cas3 levels and the Bax/Bcl‐2 ratio were attenuated by AST treatment (Figure 6G–I). Although 3‐MA alone did not affect H9c2 cells, it blocked AST's protective effects in response to alcohol treatment (Figure 6).

**FIGURE 7:**
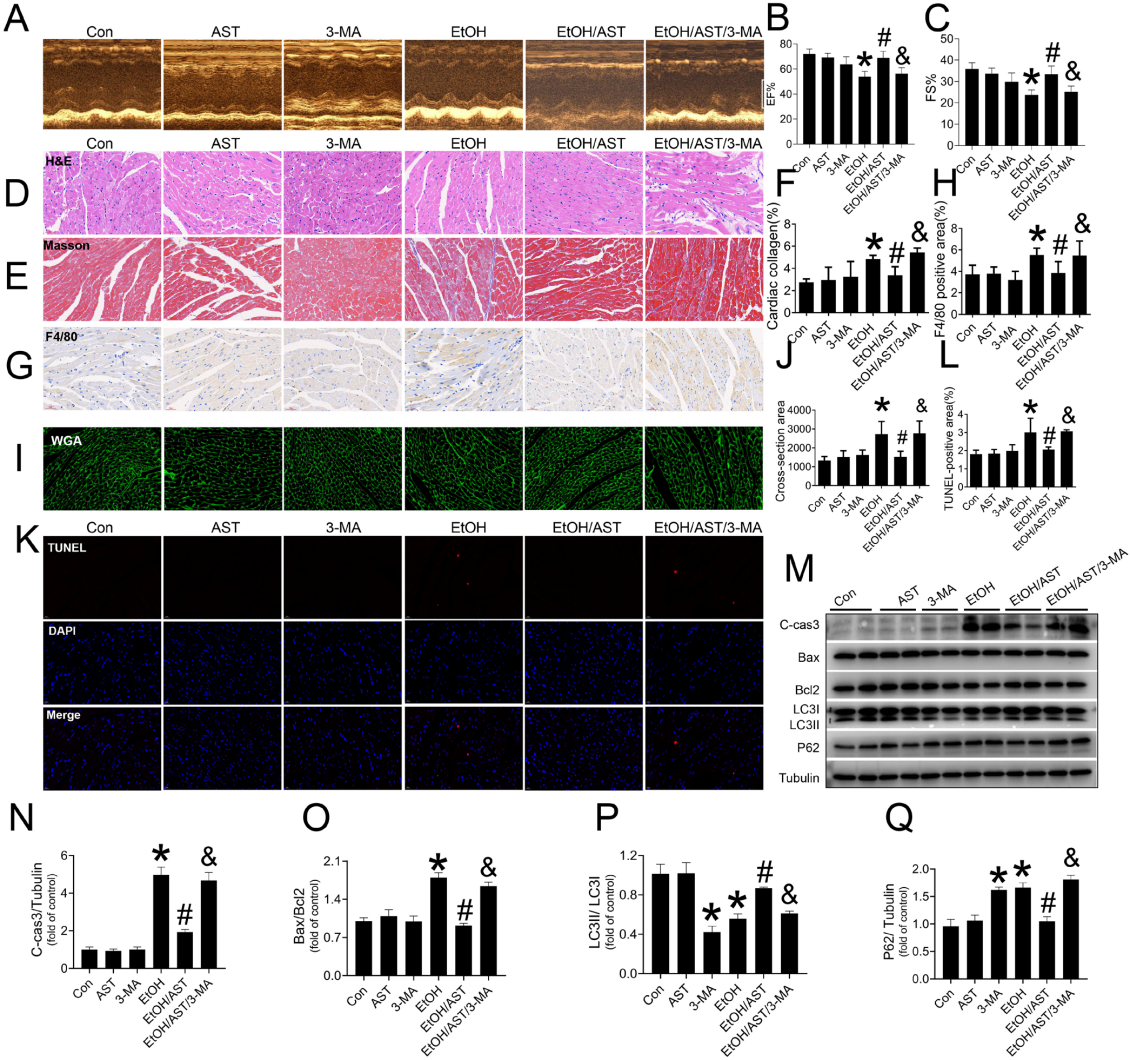
The impact of 3‐MA on AST‐induced prevention of acute ACM induced by acute alcohol treatment in C57BL/6J mice. Echocardiography was performed on mice in each group to evaluated the cardiac systolic function including %EF (A, B) and %FS (A, C). Cardiac morphological changes were detected by HE staining (D). Cardiac collagen accumulation and fibrosis were evaluated by Masson staining (E, F). Cardiac inflammation was evaluated by F4/80 staining (G, H). Cardiac hypertrophy was evaluated by WGA staining (I, J). TUNEL and its semi‐quantitative analysis was used to detected cardiac apoptosis (K, L). Western‐blot analysis was applied to detect the expression level of C‐cas3 (M, N), Bax/Bcl‐2 ratio (M, O), as well as the LC3II/I ratio (M, P) and the expression of P62 (M, Q). Data are presented as the mean ± standard deviation (*n* = 3–10/group). *p < 0.05 versus Con group; #*p* < 0.05 versus EtOH group; ^&^ *p* < 0.05 versus EtOH/AST group.

To verify the in vitro findings in vivo, C57BL/6J mice were administered 3‐MA (10 mg/kg/day) intraperitoneally for 3 days, followed by co‐treatment with AST and/or alcohol for another 5 days. The results showed that AST significantly preserved the cardiac function of mice exposed to acute alcohol treatment, as indicated by the maintenance of %EF (Figure 7A,B) and %FS (Figure 7A,C). It turned out that the protective effects of AST on cardiac function were inhibited by 3‐MA during acute alcohol treatment (Figure 7A–C). Furthermore, pretreatment with 3‐MA not only blocked AST‐induced morphological protection effect, but also offset its anti‐fibrotic, anti‐inflammatory and anti‐hypertrophic effects against acute alcohol treatment (Figure 7D–J). Meanwhile, TUNEL staining showed that 3‐MA blocked AST's anti‐apoptotic effect (Figure 7K,L), which was also confirmed by the re‐upregulated level of C‐cas3 and Bax/Bcl2 ratio (Figure 7M–O). The autophagy protective effect of AST against acute alcohol treatment was also blocked by 3‐MA, indicating that the autophagy inhibitor is efficient (Figure 7M,P,Q). These findings from both in vitro and in vivo studies indicate that AST prevents acute ACM by maintaining myocardial autophagy, probably through mitophagy, in C57BL/6J mice.

## 3 Discussion

The development of human society is closely associated with the use of alcohol, which plays a significant role in social activities [39, 40]. However, alcohol induces a series of adverse effects on drinkers, particularly health [41, 42, 43]. Several studies have demonstrated that the myocardium is a primary target organ of alcohol [44, 45, 46]. Based on the onset time and pathological characteristics, ACM can be divided into chronic and acute ACM [47, 48]. Although chronic ACM accounts for majority of cases, acute ACM is more detrimental to the myocardium in a short period [43, 47, 48]. Once excessive alcohol intake leads to a severe decrease in the contraction of cardiomyocytes and dilation of the left ventricle, it leads to heart failure and arrhythmia. This condition is associated with overt myocarditis [3, 43]. Due to acute ACM being unpredictable and difficult to treat, prevention is extremely important and prevention mainly focuses on the pathogenesis of acute ACM.

Strong evidence suggests that myocardial oxidative stress is a predominant factor in the onset of acute ACM [49, 50, 51]. Alcohol and its metabolites can disrupt the redox balance in cardiomyocytes and generate excessive ROS, which cause the oxidation of lipids, proteins and DNA in cardiomyocytes, leading to oxidative stress‐induced cardiac apoptosis [52, 53, 54]. Based on these findings, preventive drug candidates against acute ACM should possess strong antioxidative properties.

AST, with the chemical formula C_40_H_52_O_4_, is a red carotenoid ketone‐based oxygen‐containing derivative in the xanthophyll family. Several studies have indicated that AST has a an outstanding antioxidative effect and has a safe dosage window of [19, 20]. According to previous studies, oxidative stress is the connecting point between acute ACM and AST. In the present study, we first explored whether AST has a preventive effect on the development of acute ACM.

To achieve this, the precise establishment of an acute ACM model was necessary. According to a series of studies by Ren et al., C57BL/6J mice received ethanol (3 g/kg) for 5 consecutive days to establish an acute ACM model [49, 52, 55]. Subsequently, symptoms of ACM were clearly observed, including impaired cardiac systolic function, mainly characterised by reduced %EF and %FS, mitochondrial pathway‐mediated cardiac cell apoptosis and obvious cardiac oxidative stress. At the same time, hypertension and coronary plaques were not present (data not shown), indicating that cardiac injury was attributed only to alcohol treatment. It is commonly impossible to induce chronic ACM in just 5 days of alcohol treatment. Based on these results, we confirmed the successful establishment of an acute ACM mouse model.

To evaluate the preventive effect of AST on acute ACM, the mice received olive oil dissolved in AST (100 mg/kg/day) for 5 consecutive days simultaneously with acute alcohol treatment. In the present study, we found that AST administration significantly protected the cardiac function against acute alcohol treatment related to its preventive effect on cardiac morphological changes, fibrosis, inflammation, hypertrophy and cardiac dysfunction. Further studies showed that AST also protected the acute alcohol‐treated heart from oxidative stress and apoptosis, suggesting that AST prevented acute ACM associated with oxidation‐mediated cardiac cell apoptosis. Primary cardiomyocytes and H9c2 cells were used in our in vitro study, which indicated that AST could directly act on cardiomyocytes to prevent cardiomyocyte injury in response to alcohol treatment.

Mitochondrial impairment and dysfunction predominantly contribute to ROS production and the induction of oxidative stress. Additionally, in the present study, we found that AST reduced mitochondrial pathway‐mediated apoptosis induced by acute alcohol treatment, suggesting that AST administration may benefit myocardial mitochondria. Accordingly, we determined whether acute ACM is characterised by myocardial mitochondrial injury and whether the AST‐induced preventive effects on acute ACM are related to myocardial mitochondrial protection. In vitro *and* in vivo studies have demonstrated that acute alcohol treatment impairs myocardial mitochondrial function by affecting membrane potential, mitochondrial biogenesis, fusion and fission. This finding suggests that AST prevents acute ACM, probably by protecting myocardial mitochondrial function. Interestingly, the effect of acute alcohol treatment and AST treatment on OPA1 expression in vivo was not observed in the in vitro study. We suppose that the differences in dose and exposure period between in vivo and in vitro might be reasonable explanations. Additionally, according to the findings above, we also get a clue that mitochondrial fission is more sensitive to both acute alcohol and AST treatments.

Impaired myocardial mitochondrial biogenesis, fusion and fission indicate the loss of mitochondrial homeostasis which might result from the abnormal clearance of damaged mitochondria. Several studies have demonstrated that autophagy, particularly mitophagy, plays a crucial role in clearing damaged mitochondria and maintaining mitochondrial homeostasis. Our study confirmed that acute alcohol treatment impaired myocardial autophagy by decreasing the LC3II/I ratio and the expression of Beclin1 and Rab7 while increasing P62 expression. However, Tao et al. and Zhang et al. suggested that acute ethanol‐induced cardiac injury was associated with autophagy induction which contradicts our findings. In their studies, both the LC3II/I ratio and P62 expression increased simultaneously, indicating a disorder in autolysosome formation and the blockade of the entire autophagy flux rather than upregulation of autophagy. Therefore, based on their findings, it is not enough to support their conclusion. Additionally, in the present study, we used a 5‐day continuous ethanol treatment to establish acute ACM, unlike the 3‐day treatment in the studies by Tao et al. and Zhang et al. [4, 56], which might further explain the contradictory findings.

As we know, mitophagy, a form of autophagy, degrades the damaged mitochondria via lysosomes to maintain mitochondrial homeostasis. Moreover, we hypothesised that the suppression of myocardial autophagy in response to acute alcohol treatment reflects changes in mitophagy. We observed abnormal myocardial mitophagy characterised by the downregulation of the Pink‐Parkin pathway, which specifically mediates mitophagy. Furthermore, isolated mitochondrial proteins were applied in our study, which showed that the ratio of mitochondrial LC3II to LC3I was also decreased in response to acute alcohol treatment. These observations above led us to speculate that the impairment of myocardial autophagy due to acute alcohol treatment might be attributed to mitophagy impairment. The reduced mitochondrial LC3II/I ratio and the downregulated Pink‐Parkin pathway in response to acute alcohol treatment were suppressed once co‐treatment with AST, indicating that AST protects myocardial mitophagy against acute alcohol treatment.

Finally, in the mechanistic study, we used an autophagy inhibitor (3‐MA) to understand the role of myocardial autophagy, especially mitophagy, in AST‐induced preventive effects on acute ACM. In vitro and in vivo studies revealed that 3‐MA administration inhibited AST's preventive effects on acute ACM, indicating that AST prevents acute ACM by maintaining myocardial autophagy, probably by mitophagy.

## 4 Materials and Methods

### 4.1 Establishment of Acute ACM Mice Model

Eight‐week‐old male C57BL/6J mice were obtained from the Hangzhou Ziyuan Experimental Animal Technology Co. Ltd. (Zhejiang, China). They were housed under a 12‐h light–dark cycle at room temperature and given standard food. Animal care and experimental procedures were approved by the Committee on the Ethics of Animal Experiments of the Third Affiliated Hospital of Wenzhou Medical University (Ruian People's Hospital, approval ID: SYSQ‐2023‐019, Zhejiang, China).

The mice were divided into six groups including control (Con), astaxanthin (AST), 3‐methyladenine (3‐MA), alcohol (EtOH), alcohol plus astaxanthin (EtOH/AST) and alcohol plus astaxanthin plus 3‐methyladenine (EtOH/AST/3‐MA). To induce an acute ACM model, the mice received intraperitoneal injections of ethanol (3 g/kg) for five consecutive days. The control group received an equivalent volume of normal saline. To evaluate the potential impact of AST on acute ACM, both ethanol‐treated and control groups were orally administered AST (100 mg/kg/day) dissolved in olive oil. Furthermore, to investigate the role of autophagy in the preventive effects of AST against acute ACM, mice were also intraperitoneally injected with the autophagy inhibitor 3‐MA at 10 mg/kg/day concurrently with alcohol and/or AST treatment.

### 4.2 Examination of Cardiac Function

The cardiac function of the mice was evaluated following anaesthesia induction with 1.5% isoflurane (RWD Life Science Co. Ltd., Shenzhen, China). Mice were placed in a supine position on a heating pad to maintain the normal body temperature and the heart rate between 400 and 550 beats/min. The indices including left ventricle (LV) cavitary dimensions in diastole (LVID, d) and systole (LVID, s), LV end‐diastolic volume (LVEDV) and LV end‐systolic volume (LVESV) were measured using echocardiography with a Vevo 3100 ultrasound system equipped with a 30‐MHz linear array ultrasound transducer (Visual Sonics, Toronto, Canada) to calculate ejection fraction and fractional shortening.

### 4.3 Histological Analysis

Heart tissues were fixed in 4% paraformaldehyde, embedded in paraffin and sectioned into 5‐μm slices. Myocardium slices from the mice in each group were stained with Haematoxylin and eosin (H&E) to evaluate general morphological changes; with Masson to evaluate collagen accumulation (fibrosis); with F4/80 to evaluate the level of mature macrophage (inflammation); with wheat germ agglutinin (WGA) to evaluate cardiomyocyte hypertrophy; and with 8‐OhDG to evaluate oxidative damage of DNA as endogenous and exogenous factors (oxidative stress).

### 4.4 Terminal Deoxynucleotidyl Positive Cardiac End Labeling (TUNEL) Assays

TUNEL staining was used to assess apoptotic cardiac cells. Sections were treated with 4% hydrogen peroxide for 30 min, followed by proteinase K (20 mg/L) at 37°C for 15 min. After that, the slices were treated with 3% hydrogen peroxide for 5 min to inhibit the endogenous peroxidase. Then, the slices were incubated with terminal deoxynucleotidyl transferase and digoxigenin‐11‐dUTP for 15 min. Apoptotic cells were quantified by counting TUNEL‐positive cells in 10 randomly selected fields at ×40 magnification.

### 4.5 Detection of Cardiac Malondialdehyde (MDA) Production

Briefly, total proteins of cardiac tissue samples of each group were incubated in a mixture including 8.5% SDS, 25% acetic acid and 0.06% TBA at 90°C for an hour. After centrifugation at 4000 rpm, at 4°C for 15 min, the supernatant was harvested and transferred to 96‐well plates. The optical density was read at 540 nm. Data are expressed as nmol/mg protein [57].

### 4.6 Dihydroethidium (DHE) Production

The 5 mg of Dihydroethidium was dissolved in a 3.17 mL solution of DMSO to prepare a 5 mM stock solution, which was then packaged and frozen. For the cell experiment, the Dihydroethidium was diluted with PBS to a concentration of 5 μM and subsequently added dropwise into the cells in a 96‐well plate. The plate was wrapped in tinfoil and incubated at 37°C for approximately 30 min under dark conditions. After thorough washing with PBS, the cells were further incubated for about another 30 min. Upon addition of a small amount of PBS, red fluorescence could be observed under microscopy.

### 4.7 Cardiomyocyte Isolation and Culture

After anaesthetising mice with isoflurane, 0.5 cc of heparin (100 U/mL) was injected intraperitoneally and hearts were removed to isolate primary cardiomyocytes. The hearts were placed in a culture dish with 10 mL of perfusion buffer. Within 60 s, the heart aorta was attached to a Langendorff perfusion cannula and transferred to a digestion buffer with CaCl_2_ and collagenase II. The heart tissue was digested for 8 min until it was soft, white and translucent. The digestion was terminated and ventricular tissue was placed in a culture dish with a digestion solution. The cardiac tissue was torn and pipetted, and the cell suspension was transferred to a centrifuge tube with a digestion–termination solution. Cardiomyocytes were dispersed using pipettes, and recalcification yielded over 80% rod–shaped cardiomyocytes for further studies.

The H9c2 rat cardiac cell line was obtained from the Shanghai Institute of Biochemistry and Cell Biology (Shanghai, China) and cultured in high‐glucose DMEM (Gibco, Eggenstein, Germany) containing 4.5 mmol/L of D‐glucose supplemented with 10% fetal bovine serum, 100 U/mL penicillin and 100 mg/mL streptomycin in a 5% CO_2_ atmosphere.

### 4.8 ATP Assay

Following euthanasia, hearts were promptly excised, immediately flash‐frozen in liquid nitrogen and subsequently lysed. ATP concentrations in the diluted supernatant after tissue lysis were quantified using a firefly luciferase bioluminescence‐based ATP assay kit (Invitrogen, Carls‐bad, CA, USA), adhering strictly to the manufacturer's instructions. Concurrently, protein concentrations were determined using a BCA acid protein assay kit.

### 4.9 Detection of ROS Level in Heart Tissue

The heart tissue was cut into small pieces and placed in a homogeniser. Add an appropriate amount of PBS and homogenise to prepare a tissue homogenate. Take a certain amount of tissue homogenate and add it to the chemiluminescence detector. Add the lucigenin working solution to the detection cell to ensure thorough mixing with the tissue homogenate. Calculate the relative content of ROS in the mouse heart tissue based on the intensity of the chemiluminescence signal [58].

### 4.10 Isolation of Cardiac Mitochondria

Frash heart was quickly washed with 0.9% normal saline solution (NS) to remove the blood. Then, the heart was minced and homogenised in ice‐cold isolation buffer containing sucrose (250 mmol/L), mannitol (225 mmol/L), TES (5 mmol/L) and EGTA (0.3 mmol/L), pH 7.4. After centrifugation at 2000 rpm, 4°C for 5 min, the supernatant was further centrifuged at 8000 rpm, 4°C for another 5 min. The collected mitochondrial pellet was gently resuspended and incubated in respiration buffer containing sucrose (250 mmol/L), mannitol (225 mmol/L), KH_2_PO_4_ (10 mmol/L) and EGTA (1 mmol/L), pH 7.2, and the protein concentration was determined using a Multimode Reader spectrophotometer (BioTek Synergy H1, Agilent, CA, USA) [59, 60].

### 4.11 Measurement of Cardiac Mitochondrial Membrane

The isolated cardiac mitochondria were incubated with JC‐1 dye at 37°C for 30 min. The JC‐1 monomer form concentration was shown in green fluorescence (excitation at 485 nm wavelength and emission at 590 nm wavelength). The aggregate form of JC‐1 was shown in red fluorescence (excitation at 485 nm wavelength and emission at 530 nm wavelength). The ratio of red/green fluorescent intensity reflects the change of mitochondrial membrane potential.

### 4.12 Western Blot Analysis

Proteins from mouse hearts were extracted with Cell Lysis Buffer (Cell Signalling Technology, Danvers, MA, USA) with phosphatase and protease inhibitors (Roche 04906837001, Roche 05892970001, Merck Corporation, USA) and quantified using the BCA Bradford protein assay kit (Beyotime Biotech, Beijing, China) according to the manufacturer's protocols. Western blotting was performed with the following antibodies: cleaved caspase‐3 (C‐cas3, 1:1000), cleaved caspase‐9 (C‐cas9, 1:1000), B‐cell lymphoma‐2 (Bcl‐2, 1:1000), Bcl‐2 associated X protein (Bax, 1:1000), cytochrome C (CytoC, 1:1000), 4‐4‐Hydroxynonenal (HNE, 1:1000), Peroxisome proliferator‐activated receptor γ coactivator 1α (PGC‐1α, 1:1000), NF‐E2‐related factor 1 (NRF1, 1:1000), mitochondrial transcription factor A (TFAM, 1:1000), uncoupling protein 2 (UCP‐2, 1:1000), optic atrophy 1 (OPA1, 1:1000), dynamin‐related protein 1 (DRP1, 1:1000), mitochondrial fission factor (MFF, 1:1000), phosphorylated MFF (Thr146) (p‐MFF, 1:2000), phosphorylated MFF (Thr172) (p‐MFF, 1:2000), Voltage‐dependent anion‐selective channel 1/2 (VDAC1/2, 1:1000), light chain 3 (LC3, 1:1000), Sequestosome‐1 (P62, 1:1000), Beclin1 (1:1000), ras‐related protein rab‐7 (Rab7, 1:1000), Parkinson juvenile disease protein 2 (Parkin, 1:1000), ATP Synthase F1 Subunit Alpha (ATPα, 1:1000), glucose regulated protein 78kD (GRP78, 1:1000), PTEN‐induced putative kinase 1 (Pink, 1:1000) and Tubulin (1:5000) purchased from Abcam (Cambridge, MA, USA).

### 4.13 Flow Cytometry Analysis

Apoptotic H9C2 cells and primary cardiomyocytes were detected by flow cytometry (BD, NJ, USA) using an Annexin V‐FITC Apoptosis Detection Kit (KeyGEN Biotech, China) according to the manufacturer's instructions.

### 4.14 Statistical Analysis

Experimental data were processed using ImageJ software, and statistical analyses were performed using GraphPad Prism 8.0. Initially, a one‐way analysis of variance was performed to assess the differences among the various groups. Subsequently, Tukey's test was performed for detailed pairwise comparisons. Quantitative data are expressed as means ± standard error of the mean (Means ± SEM). Statistical significance was set at a *p*‐value < 0.05 and was crucial for robust result interpretation.

## 5 Conclusions

Our study confirmed that AST administration prevents acute ACM by maintaining cardiac systolic function and the normal histological structure, as well as suppressing myocardial oxidative stress‐mediated apoptosis and inflammation, fibrosis and cardiac hypertrophy. Mechanistic studies indicate that AST prevents acute ACM, likely by maintaining mitophagy‐mediated autophagy and the subsequent mitochondrial homeostasis.

## Author Contributions

**Guoyong Fan:** data curation (equal), investigation (equal), methodology (equal), validation (equal), writing – original draft (equal). **Tinghao Liu:** data curation (equal), investigation (equal), methodology (equal), resources (equal), validation (equal), writing – original draft (equal). **Xuemei Chen:** investigation (equal), methodology (equal), validation (equal). **Yan Guo:** investigation (equal), methodology (equal), validation (equal), visualization (equal). **Xuan Li:** investigation (equal), methodology (equal), validation (equal), visualization (equal). **Yue He:** methodology (equal). **Peng Hua:** investigation (equal), resources (equal), validation (equal), visualization (equal). **Xiufei Lin:** investigation (equal), methodology (equal), validation (equal), visualization (equal). **Dini Lin:** investigation (equal), methodology (equal), validation (equal), visualization (equal). **Xiaoqing Yan:** supervision (equal). **Shicui Jiang:** conceptualization (equal), formal analysis (equal), funding acquisition (supporting), project administration (equal), writing – original draft (equal). **Chi Zhang:** conceptualization (lead), funding acquisition (lead), project administration (lead), writing – review and editing (lead).

## Ethics Statement

The animal study was conducted according to the guidelines of the Declaration of Helsinki and was approved by the Wenzhou Medical University Animal Policy and Welfare Committee (Approval No.: wydw2016‐0124).

## Consent

The authors have nothing to report.

## Conflicts of Interest

The authors declare no conflicts of interest.

## Data Availability

Data are available upon reasonable request from the authors.
